# The influence of the dietary intake of vitamin C and vitamin E on the risk of gastric intestinal metaplasia in a cohort of Koreans

**DOI:** 10.4178/epih.e2022062

**Published:** 2022-07-29

**Authors:** Sung Keun Park, Yeongu Chung, Chang-Mo Oh, Jae-Hong Ryoo, Ju Young Jung

**Affiliations:** 1 Center for Cohort Studies, Total Healthcare Center, Kangbuk Samsung Hospital, Sungkyunkwan University School of Medicine, Seoul, Korea; 2 Department of Neurosurgery, Kangbuk Samsung Hospital, Sungkyunkwan University School of Medicine, Seoul, Korea; 3 Departments of Preventive Medicine, Kyung Hee University School of Medicine, Seoul, Korea; 4 Departments of Occupational and Environmental Medicine, Kyung Hee University School of Medicine, Seoul, Korea; 5 Total Healthcare Center, Kangbuk Samsung Hospital, Sungkyunkwan University School of Medicine, Seoul, Korea

**Keywords:** Antioxidant, Ascorbic acid, Vitamin E, Metaplasia

## Abstract

**OBJECTIVES:**

Studies have suggested that the dietary intake of antioxidant vitamins, such as vitamin C and vitamin E, has a potential role in inhibiting gastric carcinogenesis. The present study investigated the effect of antioxidant vitamins on the incidence of gastric intestinal metaplasia (GIM).

**METHODS:**

This study included 67,657 Koreans free of GIM who periodically underwent health check-ups. Dietary intake was assessed by a semiquantitative food frequency questionnaire based on the Korean National Health and Nutrition Examination Survey. Participants were categorized into 4 groups by quartiles of dietary vitamin C and vitamin E intake. The Cox proportional hazard assumption was used to determine the multivariable hazard ratio (HR) and 95% confidence interval (95% CI) for GIM.

**RESULTS:**

The third and fourth quartiles of vitamin C intake had a lower risk of GIM than the first quartile (multivariable-adjusted HR, 0.95; 95% CI, 0.88 to 1.03 in the second quartile, HR, 0.88; 95% CI, 0.81 to 0.97 in the third quartile, and HR, 0.85; 95% CI, 0.76 to 0.95 in the fourth quartile). Vitamin E intake greater than the second quartile level was significantly associated with a lower risk of GIM than the first quartile (multivariable-adjusted HR, 0.90; 95% CI, 0.82 to 0.97 in the second quartile, HR, 0.90; 95% CI, 0.82 to 0.99 in the third quartile, and HR, 0.83; 95% CI, 0.74 to 0.94 in the fourth quartile). This association was observed only in the subgroup analysis for men.

**CONCLUSIONS:**

Higher dietary intake of vitamin C and vitamin E was associated with a lower risk of GIM.

## INTRODUCTION

Despite the decreasing incidence of gastric cancer in developed countries, gastric cancer remains the fourth most common cancer in men and the seventh most common cancer in women [1]. Stomach cancer is most prevalent in East Asia, accounting for three-fourths of new cases worldwide [1].

The pathophysiology of gastric cancer includes sequential changes from chronic gastritis to atrophic gastritis and intestinal metaplasia, and from dysplasia to cancer [2]. Most gastric cancers occur in the setting of mucosal inflammation and are frequently preceded by the development of gastric intestinal metaplasia (GIM) [3]. Therefore, there has been increasing interest in analyzing the modifiable risk factors for GIM with the goal of preventing gastric cancer.

Epidemiological studies have suggested that a diet rich in fresh fruits and vegetables is potentially effective in reducing the risk of gastric cancer [4]. Recent studies have reported that oxidative stress and oxidant status are elevated in gastric cancer and GIM [5,6]. These reports suggest that oxidative stress and oxidants are potentially involved in the pathogenesis of GIM and gastric cancer. Fresh fruits and vegetables are rich in antioxidants including vitamin C and vitamin E. Therefore, it has been speculated that antioxidant vitamins contribute to the protective effect of fresh fruits and vegetables on gastric cancer. However, because of inconsistent findings [7,8] and the low incidence of gastric cancer [9], chemoprevention studies on gastric cancer could not guarantee the preventive effect of antioxidant vitamins against gastric cancer. Therefore, it is uncertain whether antioxidant vitamins inhibit the development of GIM.

The aim of the present study was to investigate the effect of antioxidant vitamins like vitamin C and vitamin E on the incidence of GIM. Therefore, we evaluated the risk of incident GIM according to the dietary intake of vitamin C and vitamin E. To identify the independent effect of antioxidant vitamins on GIM, we adjusted our analysis for the potential risk factors of GIM including sodium intake.

## MATERIALS AND METHODS

### Study design and participants

This study is a retrospective cohort study based on the Kangbuk Samsung Health Study (KSHS). The KSHS was a cohort study to investigate the medical data of Koreans who received periodic health check-ups in Kangbuk Samsung Hospital. The database of the KSHS included information about GIM and the intake of vitamin C and vitamin E; therefore, it was used to retrospectively analyze the risk of GIM according to the dietary intake of vitamins C and E.

We initially enrolled 149,931 subjects from the KSHS who had responded to a semiquantitative food frequency questionnaire (FFQ) and had received endoscopy between March 2011 and December 2012. For subjects who had undergone several health check-ups between March 2011 and December 2012, the first visit was regarded as the reference. We excluded 7,660 subjects with baseline GIM. Additionally, 8,339 subjects whose reported caloric intake was less than 500 kcal per day or more than 4,000 kcal per day were excluded. We further excluded 11,742 subjects with more than one missing value in the covariates and 35,709 subjects who used vitamin supplements. Finally, the baseline total of eligible study participants was 84,681. Among these, 67,657 subjects underwent repeat endoscopy between January 2013 and December 2018 and were included in the final analysis. The study participants were working-age adults with a mean age of 38.6± 6.7 years and most were employed by companies.

### Data collection: clinical, laboratory, health-related behavior, and endoscopy

Study data included a medical history based on the self-administered questionnaire, anthropometric measurements, and laboratory measurements. All study subjects were asked to respond to a health-related behavior questionnaire, which included the topics of alcohol consumption (type of alcoholic beverage and frequency of consumption), smoking (never, former, current), and exercise (frequency, duration, and intensity). The degree of physical activity was evaluated by the Korean-validated version of the International Physical Activity Questionnaire short form [10]. Diabetes mellitus (DM) was defined by the presence of more than one of the following conditions: fasting glucose ≥ 126 mg/dL, hemoglobin A1c ≥ 6.5%, or a prior diagnosis of diabetes. Hypertension was defined as a prior diagnosis of hypertension or having a measured blood pressure (BP) ≥ 140/90 mmHg. Trained nurses measured the BP in the sitting position using an automatic device (53000-E2; Welch Allyn, New York, NY, USA) 3 times after 5-minute intervals of rest. Body mass index (BMI) was calculated by dividing weight (kg) by height squared (m2). Blood samples were collected after more than 12 hours of fasting and were drawn from an antecubital vein. The fasting serum glucose was measured using the hexokinase method.

Endoscopic examinations were conducted by experienced endoscopists using a conventional white light endoscope (GIF-H260; Olympus Medical Systems, Center Valley, PA, USA). On endoscopy, gastric pathology and abnormal findings were imaged and described in terms of their location, size, and characteristics. The presence of GIM was diagnosed by endoscopic findings based on known standard criteria including white plaque-like elevations in the antrum and corpus of the stomach [11]. Examination for *Helicobacter pylori* infection was conducted as needed based on the following Korean guideline: suspected gastric cancer, mucosa-associated lymphoid tissue lymphoma, and peptic ulcer disease in the stomach and duodenum [12]. Detailed descriptions of the study population and data collection have been published in previous studies [13].

### Food frequency questionnaire data assessment

We assessed the dietary intake of KSHS participants using the FFQ that was developed for the Korean Genome Epidemiology Study. The dietary data used to design the FFQ were obtained from the Korea Health and Nutrition Examination Survey [14,15]. A detailed description of the FFQ [14] and its validation in the Korean population has been described in a previous study [15].

The food consumption frequency was composed of 9 categories (i.e., never or rarely, once a month, 2 times or 3 times a month, once or twice a week, 3 times or 4 times a week, 5 times or 6 times a week, once a day, twice a day, and 3 times a day) with 3 serving sizes for each food. Photographs of the usual food portions were included to enhance the subjects’ understanding and the reliability of the study. Total energy and nutrient intake were calculated using the Can-Pro 3.0 software developed by the Korean Nutrition Society [16]. All participants were divided into quartiles by intake of vitamin C and vitamin E.

### Outcome definitions

The entry date for the study was the date of each participant’s endoscopy as documented in their health record between March 2011 and December 2012. All participants had received health check-ups including endoscopy annually or biennially up to December 2018. Thus, follow-up was conducted from the entry date up to December 2018. If GIM was diagnosed during follow-up, the follow-up period was from the entry date to the date of GIM diagnosis. If GIM was not diagnosed up to December 2018, the follow-up period was from entry date to the last follow-up visit.

### Statistical analysis

In the 4 vitamin C and vitamin E intake quartiles, data were presented as mean± standard deviation for continuous variables and as proportions for categorical variables in the baseline study. Trend analysis of the median value of each group (based on consumption of vitamin C and vitamin E) was conducted using a linear regression model for continuous variables and the Cochran-Armitage trend test for categorical variables. For comparison of the 2 groups, the t-test was used for continuous variables and the chi-square test for categorical variables.

A Cox proportional hazards model was used to calculate the unadjusted and multivariable-adjusted hazard ratio (HR) for GIM and the 95% confidence interval (CI) in each study group (adjusted HR [95% CI]). The covariates of the multivariable model were selected from factors that could affect dietary patterns and the development of GIM. Multiple covariates included age, gender, regular exercise, BMI, smoking, alcohol intake (g/day), DM, hypertension, total caloric intake, and sodium intake. Additionally, we conducted a multivariable-adjusted analysis with multivariable models excluding sodium intake to test the effect of sodium intake on the association between antioxidant vitamins and the risk of GIM. To verify multicollinearity between variables, we analyzed the variance inflation factor (VIF) and confirmed that there were no variables with a VIF > 10. In addition, we examined interactions among covariates.

The proportional hazards assumption was confirmed by log-log plots and the Schoenfeld residual test. The incidence cases and incidence density (incidence cases per 1,000 person-years) of GIM were calculated in each group. Person-years were calculated as the time from the first visit of the subject between 2011 and 2012 to the time of the last visit or the occurrence of GIM in 2013-2018.

Subgroup analysis was conducted for gender (men and women). Additional analyses for subjects with vitamin supplementation and all subjects with or without vitamin supplementation were conducted. Because an interaction was suspected between vitamin E intake and hypertension (p= 0.012), we conducted a subgroup analysis for hypertension (subjects with hypertension and subjects without hypertension). Additionally, we analyzed the risk of GIM according to the dietary intake of vitamin A.

All statistical analyses were performed using R version 3.6.3 (R Foundation for Statistical Computing, Vienna, Austria), and a value of p-value< 0.05 (2-sided) indicated statistical significance.

### Ethics statement

Our study was approved by the Institutional Review Board (IRB) of Kangbuk Samsung Hospital (IRB No. KBSMC 2020-09-25). The IRB of Kangbuk Samsung Hospital approved the exemption of informed consent for this study because we retrospectively anonymized all data obtained from the health records.

## RESULTS

The baseline characteristics of the study population from March 2011 to December 2012 according to vitamin C and vitamin E intake quartiles are presented in Table 1. The mean age was 38.6±6.7 years, and only 0.9% (n= 620) of subjects were over 60 years old. During the follow-up period (mean, 65 months), 6.6% (n= 4,443) of subjects developed GIM. In general, women had better baseline clinical characteristics than men (Supplementary Material 1). The incidence of GIM in men was 8.3% (n= 3,678), which was significantly higher than in women (3.2%, n= 765). Subjects consuming more vitamin C and vitamin E were more likely to have favorable health behaviors such as less smoking, higher physical activity, and higher intakes of vitamin C and vitamin E. However, sodium intake proportionally increased with vitamin C and vitamin E intake. The incidence of GIM decreased with the intake of vitamin C and vitamin E.

Table 2 shows the unadjusted and the multivariable-adjusted HR and 95% CI for GIM according to the quartiles of vitamin C and vitamin E intake in all participants. The multivariable-adjusted HR and 95% CI for GIM decreased with the increase in vitamin C intake, despite a statistical insignificance in the second quartile (multivariable-adjusted HR, 0.95; 95% CI, 0.88 to 1.03 in the second quartile, HR, 0.88; 95% CI, 0.81 to 0.97 in the third quartile, and HR, 0.85; 95% CI, 0.76 to 0.95 in the fourth quartile; p for trend= 0.001). Increase in vitamin E intake was more clearly associated with the risk of GIM (second quartile multivariable-adjusted HR, 0.90; 95% CI, 0.82 to 0.97, third quartile HR, 0.90; 95% CI, 0.82 to 0.99, and fourth quartile HR, 0.83; 95% CI, 0.74 to 0.94; p for trend= 0.005).

Similar findings were observed in the subgroup analysis for men (Table 3). The third and fourth quartiles of vitamin C and vitamin E intake were significantly associated with a decreased risk of GIM, compared with the first quartile. However, subgroup analysis for women did not show a significant association between vitamin C and vitamin E intake and the risk of GIM (Table 3).

An analysis of subjects with vitamin supplementation did not show a significant association between the risk of GIM and the intake of vitamin C (Supplementary Material 2). However, vitamin E intake greater than the third quartile was associated with a decreased risk of GIM compared to the first quartile (Supplementary Material 2). In an analysis of subjects with or without vitamin supplementation, vitamin C intake greater than the third quartile was associated with a decreased risk of GIM. The intake of vitamin E showed an inverse relationship with the risk of GIM (Supplementary Material 3).

Subgroup analysis for hypertension showed that subjects without hypertension had an inverse relationship between vitamin E intake and the risk of GIM (Supplementary Material 4). However, this association was not observed in subjects with hypertension (Supplementary Material 4). The association between dietary vitamin A intake and GIM showed a similar pattern of relationship as that of vitamin C and vitamin E with GIM. While men showed a statistically significant inverse relationship between vitamin A intake and the multivariable-adjusted HR for GIM, this association was not observed in women (Supplementary Material 5). In additional analyses with multivariable models excluding sodium intake, the adjusted HR for GIM increased slightly. However, a trend of negative association between the consumption of antioxidant vitamins and the risk of GIM was found in all participants (Supplementary Material 6) and men (Supplementary Material 7). This association was not observed in women (Supplementary Material 8).

## DISCUSSION

Vitamin C and vitamin E are required nutrients that function as antioxidants. They inactivate or suppress the production of oxygen free radicals and lipid peroxidation, which have major roles in carcinogenesis [17]. Therefore, it is hypothesized that antioxidant vitamins may be involved in the intermediate process of gastric carcinogenesis. Nonetheless, the effect of antioxidant vitamins on GIM is still uncertain.

In the present study, we analyzed the risk of GIM according to the consumption of dietary vitamins C and E among 67,657 apparently healthy and relatively young Korean adults with a mean age of 38.6± 6.7 years. Our analysis showed that the intake of dietary vitamin C greater than the third quartile was associated with a decreased risk of GIM, compared with the first quartile of vitamin C intake. This association was more distinctly observed in the effect of vitamin E on GIM, which showed an inverse proportional relationship between vitamin E consumption and the risk of GIM. These results suggest the inhibitory effect of dietary antioxidant vitamins like vitamin C and vitamin E on the development of GIM.

It is widely believed that the antioxidant vitamins in fresh fruits and vegetables are effective in preventing gastric cancer. However, it is uncertain whether intake of antioxidant vitamins is effective in preventing the precancerous lesions of gastric cancer [18-22]. Our results correspond to previous reports presenting the protective effect of antioxidant vitamins against precancerous lesions including GIM [18-21]. In a randomized, controlled chemoprevention trial of 976 subjects with non-metaplastic atrophy and/or intestinal metaplasia, researchers examined the influence of vitamin C supplementation (ascorbic acid, 1 g twice a day) on gastric precancerous lesions for 72 months [18]. An analysis of 631 subjects showed that vitamin C supplementation was associated with a higher relative risk for a more rapid rate of regression in subjects with atrophy (relative risk, 5.0; 95% CI, 1.7 to 14.4) and intestinal metaplasia (relative risk, 3.1; 95% CI, 1.0 to 9.3) than the control group [18]. In a study of approximately 600 Chinese subjects with GIM, serum concentrations of ascorbic acid were significantly lower in individuals with GIM than in those with chronic atrophic gastritis (CAG) or simple gastritis [19]. Moreover, the odds ratio of CAG progressing to GIM was only one-sixth as high among those with upper tertile levels of ascorbic acid compared to those with lower tertile levels of ascorbic acid [19]. In an intervention study of 1,219 Colombians, increased intake of vitamins C and E was inversely associated with the severity of gastritis, independent of BMI, smoking status, years of education, total caloric intake, and *H. pylori* infection status [20]. Additionally, a double-blind intervention trial compared the regression rate of GIM between a high-dose vitamin E supplementation (400 units/day) group and a control group (placebo) for 12 months. While the vitamin E supplementation group showed regression in 8 of 14 subjects (57%) at 6 months and 10 of 14 subjects (71%) at 12 months, the control group did not show regression in 16 subjects [21]. However, these results are controversial. In a randomized, double-blind, chemoprevention trial to identify the effect of vitamins C and E on precancerous gastric lesions in 1,980 Venezuelans, there was no significant difference in the progression rate or regression rate of precancerous gastric lesions between the vitamin supplementation group and the control group [22]. An explanation for these conflicting results may include differences among studies in the manner of vitamin supplementation (dietary vitamin intake vs. vitamin capsules) and the vitamin dose, as well as subject ethnicity, socioeconomic status, age, and the prevalence of gastric cancer. Our study was characterized by results derived from relatively young adults, a large sample size (67,657 Koreans), dietary antioxidant vitamins, and a highly prevalent area for gastric cancer. In our study, it is assumed that a high intake of dietary antioxidant vitamins in young adults may link to good dietary habits and health behaviors. In practice, groups with higher vitamin C intake tended to have higher levels of physical activity, less smoking, and less alcohol intake. Therefore, it is postulated that high vitamin intake has a synergistic effect with good dietary habits and health behaviors in young adults. Nonetheless, the protective effect of antioxidant vitamins was maintained even after adjusting for physical intake, obesity, smoking, and alcohol intake, which suggests the independent role of antioxidant vitamins in preventing GIM.

In our analysis, men showed a protective effect of vitamins on GIM. However, this effect was not observed in women. Plausible explanations for this gender difference may include the differences in the prevalence of GIM and risk factors between men and women and the protective effect of estrogen on GIM. It has been reported that men are more predisposed to GIM than women due to higher rates of risk factors, such as *H. pylori* infection, gastric disorders, smoking, and alcohol [23,24]. Epidemiological evidence from Korea has indicated that the prevalence of GIM is lower in women than men [11]. Our men subjects also had a higher incidence of GIM and a higher proportion of smoking and alcohol use than women. Studies have demonstrated that estrogen confers resistance to gastric pathologies, including gastric cancer and GIM [25,26]. Therefore, it is inferred that the function of estrogen and fewer risk factors in women exceeds the protective effect of vitamins on GIM. Additionally, the low incidence of GIM in women may have attenuated the statistical power.

Our results suggest that the protective effect of antioxidant vitamins on GIM can vary based on other nutrients consumed along with them. When we removed the effect of sodium intake by excluding sodium intake from the adjusting covariates, the protective effect of antioxidant vitamins on GIM decreased, with a slight increase in the HR for GIM across all groups in all participants and men. Studies have demonstrated that a high salt intake is potentially associated with an increased risk of GIM [27,28]. Salt exerts harmful effects on gastric mucosa by increasing gastric carcinogens such as N-methyl-N-nitro-N-nitrosoguanidine, promoting gastric epithelial hyperplasia and parietal cell loss, and increasing *H. pylori* colonization [29,30]. To enhance the protective effect of antioxidant vitamins on GIM, consideration of co-consumed foods is recommended.

Some studies have suggested potential mechanisms underlying the protective effects of vitamin C and vitamin E on GIM. Activation of ornithine decarboxylase (ODC) is important in the pathogenesis of GIM. An intervention study found that supplementation of high dose vitamin E decreased the activity of ODC by 53% after 6 months and by 65% after 12 months [21]. The antioxidant function of vitamins C and E suppresses the production of free radicals and active oxygen species, which have an important role in gastric carcinogenesis [17]. As an efficient antioxidant at high oxygen pressure, vitamin E is an inhibitor of lipid peroxidation in cell membranes [31]. In a rodent study, vitamin C supplementation attenuated mucosal oxidative DNA damage and showed milder mucosal inflammation in short-term gastritis [32]. Vitamin C may protect against gastric carcinogenesis by scavenging mucosal reactive oxygen species or by reducing N-nitroso compound formation in the gastric juices [33]. These antioxidant functions of vitamin C and vitamin E may inhibit the development of GIM, which is a transitional step in gastric carcinogenesis.

The present study had several limitations. First, because we used endoscopic findings to diagnose GIM without an additional histological exam, there could be concerns regarding a potential mismatch of endoscopic findings with histological findings. This limitation creates the possibility of underestimation or overestimation of the endoscopic diagnosis.

Second, although *H. pylori* infection is a strong risk factor for GIM, our adjustment for covariates did not include the presence of *H. pylori* infection because testing for *H. pylori* infection was not a routine assessment in screening endoscopy in Korea. Korean endoscopic guidelines do not define GIM as an indication for testing for *H. pylori* infection [12]. Therefore, we could not identify *H. pylori* infection in all cases. These limitations warrant further studies with histological evidence for GIM and *H. pylori* infection.

Third, although the FFQ was validated for the general Korean population [15], it was not specifically validated for the KSHS study participants.

In conclusion, we found that increased consumption of dietary vitamin C and vitamin E was associated with a decreased risk of GIM in working-age Koreans. This association was more prominent in men than women. These findings provide additional evidence regarding the protective effect of antioxidant vitamins on GIM.

## Figures and Tables

**Table 1.** Baseline characteristics of the study participants according to vitamin C and vitamin E intake quartiles

Characteristics		Overall	Quartile 1	Quartile 2	Quartile 3	Quartile 4	p for trend
Vitamin C intake (n)		67,657	16,958	16,933	16,856	16,910	
	Men, gender	44,077 (65.1)	11,610 (68.5)	11,352 (67.0)	11,027 (65.4)	10,088 (59.7)	<0.001
	Age (yr)	38.6±6.7	38.8±6.9	38.4±6.5	38.5±6.6	38.8±6.9	0.926
	BMI (kg/m^2^)	23.5±3.2	23.4±3.2	23.4±3.2	23.5±3.3	23.5±3.3	<0.001
	Average alcohol use (g/day)	15.6±22.7	16.0±22.6	15.7±21.9	15.4±22.4	15.4±23.8	0.014
	Current smoker (%)	25.2	26.7	26.1	25.3	22.8	<0.001
	High PA (%)	16.8	12.9	15.5	17.2	21.7	<0.001
	DM (%)	3.4	3.6	3.3	3.2	3.5	0.572
	HTN (%)	10.2	10.7	10.3	10.0	9.9	0.011
	Vitamin C intake (mg/day)	83.2±57.7	28.9±12.2	57.8±7.3	86.9±10.1	159.6±59.7	<0.001
	Vitamin E intake (mg/day)	7.5±3.7	4.5±1.9	6.3±2.1	7.9±2.5	11.3±4.0	<0.001
	Sodium intake (mg/day)	2,158.0±1,189.3	1,211.0±602.4	3.6±0.9	4.7±1.1	7.0±2.4	<0.001
	Total calorie intake (kcal/day)	1,648.9±583.6	1,297.6±433.0	1,867.6±721.2	2,357.7±877.6	3,199.6±1,377.9	<0.001
	Intestinal metaplasia	4,443 (6.6)	1,232 (7.3)	1,122 (6.6)	1,060 (6.3)	1,029 (6.1)	<0.001
Vitamin E intake (n)		67,657	17,078	16,912	17,012	16,655	
	Men, gender	44,077 (65.1)	10,629 (62.2)	10,982 (64.9)	11,269 (66.2)	11,197 (67.2)	<0.001
	Age (yr)	38.6±6.7	39.3±7.1	38.6±6.6	38.4±6.5	38.2±6.7	<0.001
	BMI (kg/m^2^)	23.5±3.2	23.2±3.2	23.4±3.2	23.5±3.3	23.8±3.3	<0.001
	Average alcohol use (g/day)	15.6±22.7	15.3±22.6	14.9±21.5	15.5±21.5	16.9±25.0	<0.001
	Current smoker (%)	25.2	24.1	24.8	26.0	26.0	<0.001
	High PA (%)	16.8	14.2	15.7	16.7	20.7	<0.001
	DM (%)	3.4	3.7	3.2	3.5	3.3	0.140
	HTN (%)	10.2	10.7	9.8	10.1	10.3	0.361
	Vitamin C intake (mg/day)	83.2±57.7	38.5±22.4	64.5±26.9	88.4±34.5	142.9±71.2	<0.001
	Vitamin E intake (mg/day)	7.5±3.7	3.7±0.9	5.8±0.5	7.9±0.7	12.6±3.4	<0.001
	Sodium intake (mg/day)	2,158.0±1,189.3	1,323.1±755.3	1,860.8±804.7	2,278.6±900.7	3,192.9±1,336.0	<0.001
	Total calorie intake (kcal/day)	1,648.9±583.6	1,146.7±343.4	1,474.6±364.5	1,742.2±398.0	2,245.6±558.3	<0.001
	Intestinal metaplasia	4,443 (6.6)	1,263 (7.4)	1,067 (6.3)	1,102 (6.5)	1,011 (6.1)	<0.001

Values are presented as mean±standard deviation or number (%). BMI, body mass index; PA, physical activity; DM, diabetes mellitus; HTN, hypertension.

**Table 2.** Association for gastric intestinal metaplasia according to the quartiles of vitamin C and vitamin E consumption in all study participants^1^

Variables		Quartile 1	Quartile 2	Quartile 3	Quartile 4	p for trend
Vitamin C intake (n)		16,958	16,933	16,856	16,910	-
	Range of intake (mg/day)	≤45.2	42.3-70.8	70.9-105	≥107	-
	Unadjusted HR	1.00 (reference)	0.90 (0.83, 0.98)	0.85 (0.78, 0.92)	0.83 (0.76, 0.90)	<0.001
	Multivariable-adjusted HR	1.00 (reference)	0.95 (0.88, 1.03)	0.88 (0.81, 0.97)	0.85 (0.76, 0.95)	0.001
	Incidence density/person yr	13.6/90,299	12.4/90,654	11.7/90,568	11.4/90,415	-
	Incidence cases	1,232 (7.3)	1,122 (6.6)	1,060 (6.3)	1,029 (6.1)	-
Vitamin E intake (n)		17,078	16,912	17,012	16,655	-
	Range of intake (mg/day)	≤4.9	5.0-6.7	6.7-9.2	≥9.3	-
	Unadjusted HR	1.00 (reference)	0.84 (0.77, 0.91)	0.85 (0.78, 0.92)	0.80 (0.73, 0.87)	<0.001
	Multivariable-adjusted HR	1.00 (reference)	0.90 (0.82, 0.97)	0.90 (0.82, 0.99)	0.83 (0.74, 0.94)	0.005
	Incidence density/person yr	14.0/90,359	11.8/90,583	12.0/91,578	11.3/89,417	-
	Incidence cases	1,263 (7.4)	1,067 (6.3)	1,102 (6.5)	1,011 (6.1)	-

Values are presented as HR (95% confidence interval) or number (%). HR, hazard ratio.

1 Adjusted for body mass index, age, gender, physical activity, alcohol intake, smoking, hypertension, diabetes mellitus, total calorie intake, and sodium intake.

**Table 3.** Associations for gastric intestinal metaplasia according to the quartiles of vitamin C and vitamin E consumption by gender^1^

Variables			Quartile 1	Quartile 2	Quartile 3	Quartile 4	p for trend
Men							
	Vitamin C intake (n)		11,050	10,991	11,030	11,006	-
		Range of intake (mg/day)	≤44.0	44.1-68.5	68.6-102	≥102	
		Unadjusted HR	1.00 (reference)	0.91 (0.83, 1.00)	0.88 (0.80, 0.96)	0.86 (0.78, 0.94)	0.001
		Multivariable-adjusted HR	1.00 (reference)	0.95 (0.87, 1.05)	0.90 (0.82, 1.00)	0.85 (0.75, 0.96)	0.006
		Incidence density/person yr	17.0/59,092	15.5/59,223	15.0/59,579	14.6/59,138	
		Incidence cases	1,002 (9.1)	917 (8.3)	893 (8.1)	866 (7.9)	
	Vitamin E intake (n)		11,174	11,039	10,996	10,868	
		Range of intake (mg/day)	≤5.0	5.1-6.8	6.9-9.3	≥9.4	
		Unadjusted HR	1.00 (reference)	0.85 (0.78, 0.93)	0.81 (0.74, 0.89)	0.77 (0.70, 0.84)	<0.001
		Multivariable-adjusted HR	1.00 (reference)	0.93 (0.85, 1.02)	0.89 (0.80, 0.99)	0.85 (0.75, 0.97)	0.012
		Incidence density/person yr	18.0/59,470	15.4/59,382	14.8/59,628	13.9/58,553	
		Incidence cases	1,068 (7.4)	912 (6.3)	882 (6.5)	816 (6.1)	
Women							
	Vitamin C intake (n)		5,907	5,887	5,899	5,887	-
		Range of intake (mg/day)	≤47.8	47.9-75.0	75.1-115	≥115	-
		Unadjusted HR	1.00 (reference)	0.89 (0.72, 1.09)	0.89 (0.73, 1.09)	1.02 (0.84, 1.24)	0.812
		Multivariable-adjusted HR	1.00 (reference)	0.91 (0.74, 1.12)	0.88 (0.71, 1.10)	0.92 (0.72, 1.19)	0.489
		Incidence density/person yr	6.4/31,202	5.7/31,059	5.8/31,524	6.6/31,118	-
		Incidence cases	200 (3.4)	177 (3.0)	182 (3.1)	206 (3.5)	-
	Vitamin E intake (n)		6,133	525	537	5,785	-
		Range of intake (mg/day)	≤4.8	4.9-6.6	6.7-9.9	≥9.1	-
		Unadjusted HR	1.00 (reference)	0.76 (0.62, 0.92)	0.86 (0.71, 1.05)	0.83 (0.68, 1.01)	0.133
		Multivariable-adjusted HR	1.00 (reference)	0.81 (0.66, 1.00)	0.92 (0.74, 1.14)	0.80 (0.61, 1.07)	0.243
		Incidence density/person yr	7.0/32,138	5.4/31,480	6.2/30,543	5.9/30,743	-
		Incidence cases	226 (3.7)	169 (2.9)	188 (3.3)	182 (3.1)	-

Values are presented as HR (95% confidence interval) or number (%). HR, hazard ratio.

1 Adjusted for body mass index, age, physical activity, alcohol intake, smoking, hypertension, diabetes mellitus, total calorie intake, and sodium intake

## SUPPLEMENTARY MATERIALS

Supplementary Material 1. Baseline characteristics of the study participants according to the gender

Supplementary Material 2. Hazard Ratios (HRs) and 95% confidence intervals (CI) for gastric intestinal metaplasia according to the quartile groups of vitamin C and vitamin E consumption in participants with vitamin supplementary intake.

Supplementary Material 3. Hazard Ratios (HRs) and 95% confidence intervals (CI) for gastric intestinal metaplasia according to the quartile groups of vitamin C and vitamin E consumption in participants with or without vitamin supplementary intake.

Supplementary Material 4. Hazard Ratios (HRs) and 95% confidence intervals (CI) for gastric intestinal metaplasia according to the quartile groups of vitamin E consumption in participants with or without hypertension.

Supplementary Material 5. Hazard Ratios (HRs) and 95% confidence intervals (CI) for gastric intestinal metaplasia according to the quartile groups of vitamin A consumption.

Supplementary Material 6. Hazard Ratios (HRs) and 95% confidence intervals (CI) for gastric intestinal metaplasia according to the quartile groups of vitamin C and vitamin E consumption in all study participants (with covariates excluding sodium intake)

Supplementary Material 7. Hazard Ratios (HRs) and 95% confidence intervals (CI) for gastric intestinal metaplasia according to the quartile groups of vitamin C and vitamin E consumption in men (with covariates excluding sodium intake)

Supplementary Material 8. Hazard Ratios (HRs) and 95% confidence intervals (CI) for gastric intestinal metaplasia according to the quartile groups of vitamin C and vitamin E consumption in women (with covariates excluding sodium intake)
