# Supplementary material for: The influence of the dietary intake of vitamin C and vitamin E on the risk of gastric intestinal metaplasia in a cohort of Koreans
Source: Epidemiol Health. 2022 Jul 29;44:e2022062. doi: 10.4178/epih.e2022062 (PMC9754913; doi:10.4178/epih.e2022062)
Supplement: Supplementary Material 1. — Baseline characteristics of the study participants according to the gender [file epih-44-e2022062-suppl1.docx]

**Supplementary Material 1.** Baseline characteristics of the study participants according to the gender

| Characteristics | Overall | Men | Women | P value |
| --- | --- | --- | --- | --- |
| Participants (n) | 67657 | 44077 | 23580 |  |
| Age (year) | 38.6 ± 6.7 | 38.9 ± 6.8 | 38.1 ± 6.6 | <0.001 |
| BMI (kg/m^2^) | 23.5 ± 3.2 | 24.5 ± 2.9 | 21.6 ± 2.9 | < 0.001 |
| Average alcohol use (g/day) | 15.6 ± 22.7 | 20.8 ± 25.3 | 5.9 ± 11.9 | <0.001 |
| Current smoker (%) | 25.2% | 37.7% | 1.9% | <0.001 |
| High PA (%) | 16.8% | 17.2% | 16.0% | <0.001 |
| DM (%) | 3.4% | 4.4% | 1.5% | <0.001 |
| HTN (%) | 10.2% | 13.7% | 3.8% | <0.001 |
| Vitamin C intake (mg/day) | 83.2 ± 57.7 | 79.8 ± 53.4 | 89.8 ± 64.5 | <0.001 |
| Vitamin E intake (mg/day) | 7.5 ± 3.7 | 7.5 ± 3.7 | 7.4 ± 3.8 | <0.001 |
| sodium intake (g/day) | 2158.0 ± 1189.3 | 2241.0 ± 1192.1 | 2003.0 ± 1168.5 | <0.001 |
| Total calorie intake (kcal/day) | 1648.9 ± 583.6 | 1721.9 ± 583.4 | 1512.6 ± 559.0 | <0.001 |
| Intestinal metaplasia (%, [n]) | 4443 (6.6%) | 3678 (8.3%) | 765 (3.2%) | <0.001 |
| Follow-up period | 5.9/5.4 | 5.9/5.4 | 5.9/5.3 | <0.001 |

Continuous variables are expressed as mean (±SD), and categorical variables are expressed as number (percentage (%)).

BMI: body mass index, PA: Physical activity, DM: diabetes mellitus, HTN: hypertension

Follow-up period is presented as median/ mean year
