# Supplementary material for: The influence of the dietary intake of vitamin C and vitamin E on the risk of gastric intestinal metaplasia in a cohort of Koreans
Source: Epidemiol Health. 2022 Jul 29;44:e2022062. doi: 10.4178/epih.e2022062 (PMC9754913; doi:10.4178/epih.e2022062)
Supplement: Supplementary Material 2. — Hazard Ratios (HRs) and 95% confidence intervals (CI) for gastric intestinal metaplasia according to the quartile groups of vitamin C and vitamin E consumption in participants with vitamin supplementary intake. [file epih-44-e2022062-suppl2.docx]

**Supplementary Material 2.** Hazard Ratios (HRs) and 95% confidence intervals (CI) for gastric intestinal metaplasia according to the quartile groups of vitamin C and vitamin E consumption in participants with vitamin supplementary intake.

|  | **Quartile 1** | **Quartile 2** | **Quartile 3** | **Quartile 4** | **P for trend** |
| --- | --- | --- | --- | --- | --- |
| **- Vitamin C intake (n)** | 7366 | 7356 | 7376 | 7346 |  |
| Range of intake (mg/day) | ≤ 50.2 | 50.3 – 77.6 | 77.7 - 116 | ≥ 116 |  |
| Unadjusted HR | 1.00 (Reference) | 1.04 (0.91 – 1.19) | 0.95 (0.83 – 1.08) | 0.97 (0.85 – 1.11) | 0.394 |
| Multivariable-adjusted HR | 1.00 (Reference) | 1.04 (0.91 – 1.19) | 0.94 (0.81 – 1.09) | 0.90 (0.76 – 1.07) | 0.135 |
| Incidence density/person year | 11.5/38713 | 12.0/38958 | 10.9/38964 | 11.2/38611 |  |
| Incidence cases [n, (%)] | 445 (6.0%) | 468 (6.4%) | 426 (5.8%) | 433 (5.9%) |  |
| **- Vitamin E intake (n)** | 7451 | 7345 | 7402 | 7246 |  |
| Range of intake (mg/day) | ≤ 5.3 | 5.4 – 7.2 | 7.3 – 9.8 | ≥ 9.9 |  |
| Unadjusted HR | 1.00 (Reference) | 0.95 (0.84 – 1.08) | 0.85 (0.75 – 0.98) | 0.93 (0.82 – 1.06) | 0.116 |
| Multivariable-adjusted HR | 1.00 (Reference) | 0.93 (0.82 – 1.07) | 0.83 (0.71 – 0.96) | 0.83 (0.69 – 0.99) | 0.015 |
| Incidence density/person year | 12.2/39086 | 11.6/38684 | 10.5/39329 | 11.4/38148 |  |
| Incidence cases [n, (%)] | 475 (6.4%) | 450 (6.1%) | 412 (5.6%) | 435 (6.0%) |  |

Adjusted for BMI, age, sex, physical activity, alcohol intake, smoking, hypertension, DM, total calorie intake and sodium intake
