# Supplementary material for: The influence of the dietary intake of vitamin C and vitamin E on the risk of gastric intestinal metaplasia in a cohort of Koreans
Source: Epidemiol Health. 2022 Jul 29;44:e2022062. doi: 10.4178/epih.e2022062 (PMC9754913; doi:10.4178/epih.e2022062)
Supplement: Supplementary Material 3. — Hazard Ratios (HRs) and 95% confidence intervals (CI) for gastric intestinal metaplasia according to the quartile groups of vitamin C and vitamin E consumption in participants with or without vitamin supplementary intake. [file epih-44-e2022062-suppl3.docx]

**Supplementary Material 3.** Hazard Ratios (HRs) and 95% confidence intervals (CI) for gastric intestinal metaplasia according to the quartile groups of vitamin C and vitamin E consumption in participants with or without vitamin supplementary intake.

|  | **Quartile 1** | **Quartile 2** | **Quartile 3** | **Quartile 4** | **P for trend** |
| --- | --- | --- | --- | --- | --- |
| **- Vitamin C intake (n)** | 24351 | 24203 | 24284 | 24263 |  |
| Range of intake (mg/day) | ≤ 46.7 | 46.8 – 72.7 | 72.8 - 109 | ≥ 110 |  |
| Unadjusted HR | 1.00 (Reference) | 0.91 (0.85 – 0.97) | 0.90 (0.84 – 0.96) | 0.85 (0.79 – 0.91) | <0.001 |
| Multivariable-adjusted HR | 1.00 (Reference) | 0.93 (0.88 – 1.02) | 0.93 (0.86 – 1.00) | 0.86 (0.78 – 0.94) | 0.002 |
| Incidence density/person year | 13.1/129257 | 11.9/129337 | 11.8/129617 | 11.2/128971 |  |
| Incidence cases [n, (%)] | 1692 (6.9%) | 1545 (6.4%) | 1533 (6.3%) | 1445 (6.0%) |  |
| **- Vitamin E intake (n)** | 24311 | 24973 | 23965 | 23852 |  |
| Range of intake (mg/day) | ≤ 5.0 | 5.1 – 6.9 | 7.0 – 9.4 | ≥ 9.5 |  |
| Unadjusted HR | 1.00 (Reference) | 0.87 (0.81 – 0.93) | 0.85 (0.79 – 0.91) | 0.83 (0.78 – 0.89) | <0.001 |
| Multivariable-adjusted HR | 1.00 (Reference) | 0.91 (0.85 – 0.98) | 0.88 (0.81 – 0.95) | 0.84 (0.76 – 0.92) | <0.001 |
| Incidence density/person year | 13.5/128318 | 11.8/133227 | 11.5/128447 | 11.3/127191 |  |
| Incidence cases [n, (%)] | 1727 (7.1%) | 1567 (6.3%) | 1478 (6.2%) | 1443 (6.0%) |  |

Adjusted for BMI, age, sex, physical activity, alcohol intake, smoking, hypertension, DM, total calorie intake, vitamin supplementary intake and sodium intake
