# Supplementary material for: The influence of the dietary intake of vitamin C and vitamin E on the risk of gastric intestinal metaplasia in a cohort of Koreans
Source: Epidemiol Health. 2022 Jul 29;44:e2022062. doi: 10.4178/epih.e2022062 (PMC9754913; doi:10.4178/epih.e2022062)
Supplement: Supplementary Material 4. — Hazard Ratios (HRs) and 95% confidence intervals (CI) for gastric intestinal metaplasia according to the quartile groups of vitamin E consumption in participants with or without hypertension. [file epih-44-e2022062-suppl4.docx]

**Supplementary Material 4.** Hazard Ratios (HRs) and 95% confidence intervals (CI) for gastric intestinal metaplasia according to the quartile groups of vitamin E consumption in participants with or without hypertension.

|  | **Quartile 1** | **Quartile 2** | **Quartile 3** | **Quartile 4** | **P for trend** |
| --- | --- | --- | --- | --- | --- |
| **- without hypertension (n)** | 15252 | 15261 | 15296 | 14944 |  |
| Range of intake (mg/day) | ≤ 4.9 | 5.0 – 6.7 | 6.8 – 9.2 | ≥ 9.3 |  |
| Unadjusted HR | 1.00 (Reference) | 0.82 (0.75 – 0.90) | 0.83 (0.76 – 0.91) | 0.77 (0.70 – 0.84) | <0.001 |
| Multivariable-adjusted HR | 1.00 (Reference) | 0.86 (0.78 – 0.94) | 0.85 (0.77 – 0.94) | 0.76 (0.67 – 0.87) | <0.001 |
| Incidence density/person year | 13.2/81286 | 10.9/82248 | 11.1/82750 | 10.3/80665 |  |
| Incidence cases [n, (%)] | 1069 (7.0%) | 897 (5.9%) | 915 (6.0%) | 828 (5.5%) |  |
| **- with hypertension (n)** | 1740 | 1737 | 1716 | 1711 |  |
| Range of intake (mg/day) | ≤ 4.8 | 4.9 – 6.7 | 6.8 – 9.2 | ≥ 9.3 |  |
| Unadjusted HR | 1.00 (Reference) | 0.93 (0.76 – 1.15) | 0.97 (0.79 – 1.19) | 0.96 (0.78 – 1.17) | 0.758 |
| Multivariable-adjusted HR | 1.00 (Reference) | 1.08 (0.87 – 1.34) | 1.17 (0.93 – 1.48) | 1.23 (0.93 – 1.63) | 0.112 |
| Incidence density/person year | 21.5/8658 | 20.3/8749 | 21.2/8828 | 20.9/8752 |  |
| Incidence cases [n, (%)] | 186 (7.1%) | 178 (6.3%) | 187 (6.2%) | 183 (6.0%) |  |

Adjusted for BMI, age, sex, physical activity, alcohol intake, smoking, DM, total calorie intake, vitamin supplementary intake and sodium intake
