# Supplementary material for: The influence of the dietary intake of vitamin C and vitamin E on the risk of gastric intestinal metaplasia in a cohort of Koreans
Source: Epidemiol Health. 2022 Jul 29;44:e2022062. doi: 10.4178/epih.e2022062 (PMC9754913; doi:10.4178/epih.e2022062)
Supplement: Supplementary Material 5. — Hazard Ratios (HRs) and 95% confidence intervals (CI) for gastric intestinal metaplasia according to the quartile groups of vitamin A consumption. [file epih-44-e2022062-suppl5.docx]

**Supplementary Material 5.** Hazard Ratios (HRs) and 95% confidence intervals (CI) for gastric intestinal metaplasia according to the quartile groups of vitamin A consumption.

|  | **Quartile 1** | **Quartile 2** | **Quartile 3** | **Quartile 4** | **P for trend** |
| --- | --- | --- | --- | --- | --- |
| **- All participants (n)** | 16920 | 16914 | 16909 | 16914 |  |
| Range of intake (R.E.) | ≤ 226 | 226 – 332 | 332 – 488 | ≥ 488 |  |
| Unadjusted HR | 1.00 (Reference) | 0.87 (0.80 – 0.94) | 0.89 (0.82 – 0.96) | 0.95 (0.87 – 1.03) | 0.251 |
| Multivariable-adjusted HR | 1.00 (Reference) | 0.89 (0.82 – 0.97) | 0.90 (0.82 – 0.99) | 0.88 (0.79 – 0.99) | 0.045 |
| Incidence density/person year | 13.2/89969 | 11.6/90969 | 11.8/90929 | 12.6/90069 |  |
| Incidence cases [n, (%)] | 1187 (7.0%) | 1051 (6.2%) | 1072 (6.3%) | 1133 (6.7%) |  |
| **- Men (n)** | 11027 | 11020 | 11016 | 11014 |  |
| Range of intake (R.E.) | ≤ 229 | 229 – 335 | 335 – 489 | ≥ 489 |  |
| Unadjusted HR | 1.00 (Reference) | 0.84 (0.77 – 0.92) | 0.84 (0.76 – 0.92) | 0.87 (0.80 – 0.96) | 0.004 |
| Multivariable-adjusted HR | 1.00 (Reference) | 0.87 (0.79 – 0.96) | 0.86 (0.78 – 0.96) | 0.81 (0.71 – 0.92) | 0.002 |
| Incidence density/person year | 17.4/58925 | 14.7/59593 | 14.6/59603 | 15.3/58912 |  |
| Incidence cases [n, (%)] | 1026 (9.3%) | 877(8.0%) | 873 (7.9%) | 902 (8.2%) |  |
| **- Women (n)** | 5897 | 5898 | 5890 | 5895 |  |
| Range of intake (R.E.) | ≤ 229 | 229 – 335 | 335 – 489 | ≥ 489 |  |
| Unadjusted HR | 1.00 (Reference) | 0.93 (0.75 – 1.15) | 1.04 (0.84 – 1.27) | 1.27 (1.05– 1.55) | 0.007 |
| Multivariable-adjusted HR | 1.00 (Reference) | 0.95 (0.77 – 1.19) | 1.07 (0.85 – 1.34) | 1.24 (0.95 – 1.62) | 0.082 |
| Incidence density/person year | 5.7/31017 | 5.4/31518 | 6.0/31239 | 7.4/31129 |  |
| Incidence cases [n, (%)] | 178 (3.0%) | 170 (2.9%) | 187(3.2%) | 230 (3.9%) |  |

Adjusted for BMI, age, sex, physical activity, alcohol intake, smoking, DM, total calorie intake, and sodium intake
