# Supplementary material for: The influence of the dietary intake of vitamin C and vitamin E on the risk of gastric intestinal metaplasia in a cohort of Koreans
Source: Epidemiol Health. 2022 Jul 29;44:e2022062. doi: 10.4178/epih.e2022062 (PMC9754913; doi:10.4178/epih.e2022062)
Supplement: Supplementary Material 6. — Hazard Ratios (HRs) and 95% confidence intervals (CI) for gastric intestinal metaplasia according to the quartile groups of vitamin C and vitamin E consumption in all study participants (with covariates excluding sodium intake) [file epih-44-e2022062-suppl6.docx]

**Supplementary Material 6.** Hazard Ratios (HRs) and 95% confidence intervals (CI) for gastric intestinal metaplasia according to the quartile groups of vitamin C and vitamin E consumption in all study participants (with covariates excluding sodium intake)

|  | **Quartile 1** | **Quartile 2** | **Quartile 3** | **Quartile 4** | **P for trend** |
| --- | --- | --- | --- | --- | --- |
| **- Vitamin C intake** | 16958 | 16933 | 16856 | 16910 |  |
| Range of intake (mg/day) | ≤ 45.2 | 42.3 – 70.8 | 70.9 - 105 | ≥ 107 |  |
| Unadjusted HR | 1.00 (Reference) | 0.90 (0.83 – 0.98) | 0.85 (0.78 – 0.92) | 0.83 (0.76 – 0.90) | <0.001 |
| Multivariable-adjusted HR | 1.00 (Reference) | 0.97 (0.89 – 1.05) | 0.92 (0.84 – 1.00) | 0.91 (0.83 – 1.00) | 0.026 |
| Incidence density/person year | 13.6/90299 | 12.4/90654 | 11.7/90568 | 11.4/90415 |  |
| Incidence cases [n, (%)] | 1232 (7.3%) | 1122 (6.6%) | 1060 (6.3%) | 1029 (6.1%) |  |
| **- Vitamin E intake** | 17078 | 16912 | 17012 | 16655 |  |
| Range of intake (mg/day) | ≤ 4.9 | 5.0 – 6.7 | 6.7 – 9.2 | ≥ 9.3 |  |
| Unadjusted HR | 1.00 (Reference) | 0.84 (0.77 – 0.91) | 0.85 (0.78 – 0.92) | 0.80 (0.73 – 0.87) | <0.001 |
| Multivariable-adjusted HR | 1.00 (Reference) | 0.91 (0.83 – 0.99) | 0.92 (0.84 – 1.00) | 0.87 (0.78 – 0.97) | 0.019 |
| Incidence density/person year | 14.0/90359 | 11.8/90583 | 12.0/91578 | 11.3/89417 |  |
| Incidence cases [n, (%)] | 1263 (7.4%) | 1067 (6.3%) | 1102 (6.5%) | 1011 (6.1%) |  |

Adjusted for BMI, age, sex, physical activity, alcohol intake, smoking, hypertension, DM, total calorie intake
