# Supplementary material for: The influence of the dietary intake of vitamin C and vitamin E on the risk of gastric intestinal metaplasia in a cohort of Koreans
Source: Epidemiol Health. 2022 Jul 29;44:e2022062. doi: 10.4178/epih.e2022062 (PMC9754913; doi:10.4178/epih.e2022062)
Supplement: Supplementary Material 7. — Hazard Ratios (HRs) and 95% confidence intervals (CI) for gastric intestinal metaplasia according to the quartile groups of vitamin C and vitamin E consumption in men (with covariates excluding sodium intake) [file epih-44-e2022062-suppl7.docx]

**Supplementary Material 7.** Hazard Ratios (HRs) and 95% confidence intervals (CI) for gastric intestinal metaplasia according to the quartile groups of vitamin C and vitamin E consumption in men (with covariates excluding sodium intake)

|  | **Quartile 1** | **Quartile 2** | **Quartile 3** | **Quartile 4** | **P for trend** |
| --- | --- | --- | --- | --- | --- |
| **- Vitamin C intake (n)** | 11050 | 10991 | 11030 | 11006 |  |
| Range of intake (mg/day) | ≤ 44.0 | 44.1 – 68.5 | 68.6 - 102 | ≥ 102 |  |
| Unadjusted HR | 1.00 (Reference) | 0.91 (0.83 – 0.996) | 0.88 (0.80 – 0.96) | 0.86 (0.78 – 0.94) | 0.001 |
| Multivariable-adjusted HR | 1.00 (Reference) | 0.97 (0.89 – 1.06) | 0.93 (0.85 – 1.03) | 0.89 (0.81 – 0.997) | 0.033 |
| Incidence density/person year | 17.0/59092 | 15.5/59223 | 15.0/59579 | 14.6/59138 |  |
| Incidence cases [n, (%)] | 1002 (9.1%) | 917 (8.3%) | 893 (8.1%) | 866 (7.9%) |  |
| **- Vitamin E intake (n)** | 11174 | 11039 | 10996 | 10868 |  |
| Range of intake (mg/day) | ≤ 5.0 | 5.1 – 6.8 | 6.9 – 9.3 | ≥ 9.4 |  |
| Unadjusted HR | 1.00 (Reference) | 0.85 (0.78 – 0.93) | 0.81 (0.74 – 0.89) | 0.77 (0.70 – 0.84) | <0.001 |
| Multivariable-adjusted HR | 1.00 (Reference) | 0.94 (0.85 – 1.03) | 0.90 (0.82 – 0.998) | 0.88 (0.78 – 0.99) | 0.025 |
| Incidence density/person year | 18.0/59470 | 15.4/59382 | 14.8/59628 | 13.9/58553 |  |
| Incidence cases [n, (%)] | 1068 (7.4%) | 912 (6.3%) | 882 (6.5%) | 816 (6.1%) |  |

Adjusted for BMI, age, physical activity, alcohol intake, smoking, hypertension, DM, total calorie intake
