# Supplementary material for: The influence of the dietary intake of vitamin C and vitamin E on the risk of gastric intestinal metaplasia in a cohort of Koreans
Source: Epidemiol Health. 2022 Jul 29;44:e2022062. doi: 10.4178/epih.e2022062 (PMC9754913; doi:10.4178/epih.e2022062)
Supplement: Supplementary Material 8. — Hazard Ratios (HRs) and 95% confidence intervals (CI) for gastric intestinal metaplasia according to the quartile groups of vitamin C and vitamin E consumption in women (with covariates excluding sodium intake) [file epih-44-e2022062-suppl8.docx]

**Supplementary Material 8.** Hazard Ratios (HRs) and 95% confidence intervals (CI) for gastric intestinal metaplasia according to the quartile groups of vitamin C and vitamin E consumption in women (with covariates excluding sodium intake)

|  | **Quartile 1** | **Quartile 2** | **Quartile 3** | **Quartile 4** | **P for trend** |
| --- | --- | --- | --- | --- | --- |
| **- Vitamin C intake (n)** | 5907 | 5887 | 5899 | 5887 |  |
| Range of intake (mg/day) | ≤ 47.8 | 47.9 – 75.0 | 75.1 - 115 | ≥ 115 |  |
| Unadjusted HR | 1.00 (Reference) | 0.89 (0.72 – 1.09) | 0.89 (0.73 – 1.09) | 1.02 (0.84 – 1.24) | 0.812 |
| Multivariable-adjusted HR | 1.00 (Reference) | 0.93 (0.76 – 1.15) | 0.92 (0.75 – 1.15) | 1.01 (0.81 – 1.14) | 0.990 |
| Incidence density/person year | 6.4/31202 | 5.7/31059 | 5.8/31524 | 6.6/31118 |  |
| Incidence cases [n, (%)] | 200 (3.4%) | 177 (3.0%) | 182 (3.1%) | 206 (3.5%) |  |
| **- Vitamin E intake (n)** | 6133 | 5925 | 5737 | 5785 |  |
| Range of intake (mg/day) | ≤ 4.8 | 4.9 – 6.6 | 6.7 – 9.9 | ≥ 9.1 |  |
| Unadjusted HR | 1.00 (Reference) | 0.76 (0.62 – 0.92) | 0.86 (0.71 – 1.05) | 0.83 (0.68 – 1.01) | 0.133 |
| Multivariable-adjusted HR | 1.00 (Reference) | 0.83 (0.68 – 1.03) | 0.96 (0.77 – 1.19) | 0.88 (0.67 – 1.14) | 0.533 |
| Incidence density/person year | 7.0/32138 | 5.4/31480 | 6.2/30543 | 5.9/30743 |  |
| Incidence cases [n, (%)] | 226 (3.7%) | 169 (2.9%) | 188 (3.3%) | 182 (3.1%) |  |

Adjusted for BMI, age, physical activity, alcohol intake, smoking, hypertension, DM, total calorie intake
